# Quality and cost outcomes of an integrated supportive care program

**DOI:** 10.1007/s00520-021-06450-z

**Published:** 2021-08-01

**Authors:** Elizabeth Weinstein, Matthew Kemmann, Sara L. Douglas, Barbara Daly, Nathan Levitan

**Affiliations:** 1 Cleveland Clinic Foundation, Cleveland, OH USA; 2 University Hospitals of Cleveland, Cleveland, OH USA; 3 Case Western Reserve University, Cleveland, OH USA

**Keywords:** Palliative care, Care coordination, Supportive care, Cancer care

## Abstract

**Purpose:**

This article reports findings from a demonstration project funded by the Center for Medicare and Medicaid Innovation (CMMI). The purpose of the project was to test a supportive care program on the outcomes of quality of care and quality of life, and costs in patients with advanced cancer.

**Methods:**

The project was conducted between February 2015 and February 2018, enrolling adult, Medicare or Medicaid beneficiaries with advanced or progressed solid tumor malignancy. A comparative longitudinal comparison of the program with both a concurrent control and an historic control was used to evaluate outcomes. The intervention included routine electronic biopsychosocial screening, early access to specialty palliative care, and nurse care coordination. Quality of life, aggressiveness of care, and healthcare utilization were measured.

**Results:**

A total of 1340 people were enrolled, with 71% of the total sample being Caucasian; 41.4% had stage IV cancer, and 20% utilized Medicaid only. Significant differences in the enrolled patients and the comparison group were controlled for through statistical analysis. There were significantly fewer ED visits, unplanned admissions, and fewer total hospitalization days in the intervention group. In the last 30 days of life, hospital and ICU admissions were less and a greater proportion of patients were enrolled in hospice in the intervention group. Quality of life had a marked improvement for enrolled patients. Average cost per member per month was not less in the enrolled group.

**Conclusion:**

This pragmatic demonstration project confirmed the clinical benefits of an integration of supportive care for patients with advanced cancer, although no reduction in costs was found.

The body of literature devoted to the impact of palliative care on healthcare systems and the patients and communities they serve has been accelerating over the last decade. An increasing amount of data suggests that palliative care improves clinical outcomes. While much of the established data show value is weighted towards end-of-life care, recent studies also suggest that earlier palliative care produces favorable impacts, even over late palliative care, leading to a formal endorsement by the Institute of Medicine and the American Society of Clinical Oncology [1–4]. Much of the established outcomes data have focused on reduced symptom burden and improved quality of life [5–8], but newer data increasingly suggests that palliative care may have merit in reducing healthcare utilization and cost [9, 10]. Early studies focused on cost have led the Institute for Clinical and Economic Review to estimate that specialized palliative care, at scale, could produce a favorable economic impact [11].

Despite these encouraging data, healthcare systems have been slow to fully incorporate palliative care as part of routine integrated approaches. There are a number of possible explanations for this [12]. First, reimbursement policy significantly lags in fully paying for supportive care resources such as palliative specialists, music therapy, acupuncture, and other integrative therapies. Adoption and expansion of palliative care also is constrained by provider shortages, misconceptions about the demand for and role or value of palliative care as part of standard treatment. Efforts to document the cost effectiveness of palliative care and compare outcomes across studies have been hampered by variability in the characteristics of each institution’s patient population and lack of risk stratification.

The Center for Medicare and Medicaid Innovation (CMMI), authorized under the Affordable Care Act and seeded with a $10B initial investment, was created to accelerate the science — and more importantly its application — of smarter healthcare spending [13]. Since its inception in 2010, CMMI has launched more than 80 new demonstration projects, intending to directly address our nation’s concerns about the growth in aggregate healthcare expenditures. Initially, among the wide variety of models which primarily focused on Accountable Care Organizations (ACOs), medical homes, or bundled-care programs, oncology care largely escaped scrutiny. This has changed recently, as the healthcare industry has increasingly recognized the high cost of cancer and its impact on both ACO performance and aggregate national expenditures.

In 2015, the Seidman Cancer Center of University Hospitals Cleveland Medical Center was awarded a CMMI Innovations grant (1C1CMS331349). The purpose of our program, known internally as “LINCC” (Learning Individual Needs and Coordinating Care), was to achieve the healthcare “triple aim” — improved quality, enhanced patient experience and quality of life, and reduced total costs. The program targeted the most complex segment of our cancer population — those with the most advanced cancer with or without complicating clinical or psychosocial factors which contribute to poorer clinical outcomes and higher cost. We report here on the project and selected outcomes.

## Methods

### Design

The LINCC project was conducted as a demonstration program in northeast Ohio operating between February 2015 and February 2018. This program was funded in part by the CMMI, with some additional in-kind funding provided by University Hospitals (UH). A longitudinal comparison of the program with both a concurrent control and an historic control was used to evaluate outcomes.

Patient enrollment occurred over two and a half years from one of four sites (one main campus and three community locations) within the UH Seidman network. Concurrent control patients were selected from candidates who were identified as eligible but not enrolled because of staffing constraints. This control sample was used to evaluate the impact of our intervention on patient experience, utilization, and cost outcomes, including end-of-life measures. Since quality of life data were not available across the entire cancer center, we used an historic control population for evaluating the program’s impact on quality of life. The historic control was identified from a prior NCI-funded trial performed at the same institution from 2008 to 2011 (NIH NRO18717).

The study project was deemed to be exempt, as a federal demonstration project, by the institutional review board at University Hospitals Cleveland Medical Center. Thus, written informed consent was not required.

### Sample

Eligibility criteria included the following: (1) 18 years of age or older; (2) Medicare or Medicaid beneficiary, and (3) advanced or progressed solid tumor malignancy. Patients who were beneficiaries of commercial insurance were ineligible, unless the commercial insurance was secondary.

Eligible patients were identified by a variety of means, such as tumor board reports, appointment schedules, case reviews of the electronic medical record (EMR), and referrals from the primary oncology teams. The identification of potential candidates for the program was performed by an administrative member of the team with clinical experience. Once identified, one of the nurse care coordinators approached the patient, explained the program, and collected baseline data during that initial patient assessment of needs. Patients identified as clinically eligible but who were unable to be enrolled due to program capacity provided the concurrent control group.

### Intervention

The intervention included three primary components: routine electronic biopsychosocial screening, early access to specialty-level palliative care providers, and nurse care coordination. We developed and used an electronic, tablet-based tool for obtaining patient-reported symptoms and concerns. Patients were screened at routine intervals, monthly during scheduled clinic visits or, if they were not being seen monthly, at midpoint between visits to their oncologist. They underwent a monthly, short screen which included the Edmonton Symptom Assessment Scale (ESAS), Distress Thermometer, and Problem Checklist. Upon enrollment and at months 3, 6, 15, and 24, enrollees completed an extended screen which incorporated the monthly screen and additional measures, including the Functional Assessment of Cancer Therapy (FACT-G). The monthly screens were focused on identifying symptoms which warranted immediate attention, while the extended screen was done for the purpose of gathering more comprehensive data on overall well-being. Nurses reviewed the responses and directed clinically actionable concerns to the patient’s providers, including palliative care providers as appropriate.

A second component incorporated in the program was early referrals to specialty-level palliative care in the outpatient setting. The palliative care providers (MD or nurse practitioner) coordinated with the primary oncology team for both symptom management and advance care planning. The program’s original design was for all enrolled patients to see a palliative care provider. In the first year of the program, only about 60% of enrollees participated in an early consultation with palliative care. This rate was a function of supporting patient choice of whether or not to accept referral to palliative care providers, as well as an attempt to refer all patients in the program to early palliative care, even those with low symptom burden. As the program increased capacity, demonstrated capability and value to the primary oncology teams, and improved the enrollment process to focus increasingly on higher-risk patients, 98% of patients enrolled in the last year of the program participated in early palliative care consults.

The final primary component of the program was providing nurse care coordination for these complex patients. Nurses were aligned with patients in the same way that other supportive care team members (e.g., social workers) are aligned with disease teams within the cancer center. This design was to facilitate acceptance of and ease of communication by the project nurses with the oncology teams and increase the care coordinators’ familiarity with treatment protocols.

Nurse care coordinators on the project also served as the nursing support for the palliative care providers. Coordinators were responsible for tracking the biopsychosocial screening schedule of the patients and contacted the patients for assessment either by telephone or in person (if patient was coming to the cancer center). They connected the patient to any intervention or resources which the screening identified as a need. Nurses tracked admissions and emergency department (ED) visits within our hospital system for enrolled patients and facilitated appropriate follow-up. In doing so, they assured that the primary oncology team was notified of the admission or ED visit. If the patient had not yet been seen by a palliative care provider, they facilitated referral to either the inpatient or outpatient team, as appropriate. In addition, the nurse care coordinators provided clinical follow-up for issues generated by the supportive oncology/ palliative care interdisciplinary team.

The program, through its development of new infrastructure for electronic patient biopsychosocial screening, expansion of specialty-level palliative care providers, and use of nurse care coordinators, gradually became integrated into the fabric of the cancer center. The project team members participated in work to sustain and spread the program within the organization. This included multiple educational sessions for staff, a midpoint survey of oncologists to assess their satisfaction with the program and solicit input on how to improve it, and design and implementation of a comprehensive electronic medical record addition to document end-of-life and goals of care discussions.

### Measures

Quality of life was measured using the Functional Assessment of Cancer Therapy-General (FACT-G) which is a 27-item screen validated in patients with any form of cancer which measures the four primary QOL domains: Physical Well-Being, Social/Family Well-Being, Emotional Well-Being, and Functional Well-Being [14]. The FACT-G asks patients to report upon how they felt in the past 7 days on a five-point Likert scale from “Not at All” to “Very Much.” For our use, the tool was built within a website and either given to patients on a tablet to fill out or read by the nurse coordinators to the patients over the phone and the nurses entered the responses. The FACT-G was the measure of quality of life incorporated as part of the extended screen, collected at baseline and months 3, 6, 15, and 24 of enrollment.

Aggressiveness of care was measured with multiple metrics, categorized into end-of-life measures and active treatment. End-of-life measures included death in the hospital, death in the ICU, ICU admission in the last 30 days of life, hospitalization in the last 30 days of life, emergency department visit in the last 30 days of life, chemotherapy in the last 14 days of life, rates of hospice admission, hospice length of stay, and healthcare costs within the last 30 and 90 days of life. Active treatment measures included normalized rates, on a per member per month basis, of inpatient admissions (emergent, urgent, and total), total patient days, patient days in excess of CMS MSDRG geometric length of stay, emergency department visits, and total cost. These indicators have been commonly used to reflect aggressiveness of care at the end of life [15, 16].

Healthcare utilization data were extracted from an internal financial decision support system providing detailed billing data for claims paid to wholly owned University Hospitals’ entities. This source did not include access to oral pharmaceutical costs and acute healthcare utilization outside of the University Hospitals network. Full claims datasets from CMS for similar populations at UH suggest that the costs outside our wholly owned entities are about 10% of total claims. Hospice utilization data, including admission dates and length of stay, were obtained through a partnership with two hospice programs which combined represented 85% of hospice admissions from our system.

### Analysis

SPSS Version 24.0 was used for all analyses with *p* < 0.05 used as the criterion for statistical significance. Linear regressions were conducted for all cost analyses. Data met the assumptions for linear regression and no transformations were required. Estimated marginal means were used for comparison. Prior to conducting analyses, outlier cases whose average cost per member per month was > 2.5 standard deviations above the total group mean were removed. This represented 13.2% of total cases (*n* = 215 LINCC and *n* = 113 control). Next, covariates were entered in step 1 with the grouping variable (LINCC vs control) entered in step 2. The following covariates were included in the analyses because there were statistically significant differences between groups (see Table 1): patient race, patient Medicaid status, cancer type, cancer stage, treatment status, weighted Hierarchical Condition Categories (HCC) system code, and number of palliative care visits. The HCC risk adjustment methodology is used by CMS to predict healthcare costs, establish Medicare advantage rates, and adjust performance calculations for value-based models such as Accountable Care Organizations (ACOs) [17].

**Table 1:** Sample characteristics

Variable	Not enrolled (*n* = 1138)	Enrolled (*n* = 1340)	*p*-value
Race: Caucasian	77.9%	65.8%	< .001
Medicaid only: Yes	17.7%	22.5%	< .001
Type of cancer			< .001
• GI	21.3%	26.8%	
• Thoracic	12.4%	22.8%	
• H&N	8.3%	14.1%	
• Breast	16.9%	10.3%	
• Urology	12.1%	7.1%	
CA stage IV	23.7%	56.5%	< .001
Treatment:			.016
• None	35.6%	31.9%	
• Chemo + XRT + OR^a^	5.6%	8.1%	
• All others	58.8%	60.0%	
Palliative care:			< .001
• 0–1 Visit	96.9%	62.8%	
• > 1 Visit	3.1%	37.2%	
HCC weighted^b^	1.72 (1.4)	2.83 (1.5)	< .001

^a^*Chemo*, chemotherapy; *XRT*, radiation therapy; *OR*, surgical resection

^b^HCC weighted = Medicare Hierarchical Condition Categories

For non-cost-related analyses (hospice, palliative care), there were no cost outliers removed for analytic purposes. Chi-square analyses were employed for categorical analyses, analysis of variance analyses were employed for continuous analyses, and linear regression was used for analyses that employed covariates. In order to examine the association of palliative care with hospice use patterns, palliative care visits were dichotomized into 2 categories: 0–1 visits and > 1 visits. When examining the total number of palliative care visits per patient (which ranged from 0 to 25), the 75th percentile was 1.0 visit, thus guiding the decision to group palliative care visits.

For comparison of quality of life scores over time, using the Functional Assessment of Cancer Therapy-General (FACT-G) form, repeated measures ANOVA was used.

## Results

### Sample

The demographic characteristics of the sample are shown in Table 1. Consistent with the hospital’s population, the total sample was 71% Caucasian. Twenty percent of the sample utilized Medicaid as the only health insurance. Gastrointestinal cancers were the most common diagnosis. One-third of the participants were receiving no cancer-directed therapy during the study period; such patients had likely successfully completed therapy or may have exhausted all cancer-directed treatment options. Although patients in both groups all had advanced cancer, there were significant differences in many variables when comparing patients enrolled in the LINCC project and those not enrolled, and these were controlled through statistical analysis.

### Outcomes

Table 2 presents the total average cost of care during the study period. Cost, emergency room visits, and admissions were normalized to “per member per month” in both cohorts. Resource use in the periods prior to death (30 days and 90 days) is shown for patients who died during the study period.

**Table 2:** Resource use among control and LINCC (CMMI) subjects

**Variable**^**a**^	**Control (*n* = 1025)**	**LINCC (*n* = 1125)**	***P***	**Variables with significant standardized beta**^**b**^
Ave cost PMPM^c^	$3,658	$4,045	.06	HCC, Race, Medicaid, Type CA, Tx Cat (3), Pall Care
Emergency room visits	0.141	0.118	.052	HCC, Pall Care, Race, Type CA
Admissions, total	0.114	0.101	.212	Race, Tx Cat (3), Pall Care, Type CA, Ca Stage
Admissions, urgent or emergent	1.00	0.77	.004	HCC, Ca Stage, Race, Pall Care, Type CA, Tx Cat (3)
Total inpatient days	6.94	4.46	< .001	HCC, Pall Care, Stage, Race, Type CA, Tx Cat (3)
**EOL variables**	**(*n* = 357)**	**(*n* = 614)**		
Cost in last 30 days	$12,680	$9,837	.08	Race, Tx Cat (3), Type CA
Cost in last 3 months	$21,466	$16,871	.004	Medicaid, HCC, Tx Cat (3), Pall Care
Chemotherapy encounters in last 14 days	0.033	0.036	.86	Tx Cat (3)
Emergency room encounters in last 30 days	0.48	0.33	.004	HCC, Type Ca, Race
Admissions in last 30 days	0.60	0.32	< .001	HCC
Admissions to intensive care in last 30 days	0.15	0.04	< .001	

^a^Cost outliers (Ave cost PMPM) removed if ≥ 2.5 SD from sample mean

^b^Covariates: race (1 = Caucasian, 2 = non Caucasian), Medicaid only (0 = not Medicaid, 1 = Medicaid), cancer type (8 categories), stage (0 = stages 1–3, 1 = stage 4), Tx Cat (0 = no treatment, 1 = all 3 treatments [chemotherapy, radiation, surgery], 2 = all other variations of 1 or 2 treatments), weighted Hierarchical Condition Categories [HCC], palliative care visits (0 = 0–1 visits, 1 =  > 1 visit). All means are estimated marginal means

^c^*PMPM*, per member, per month

As can be seen, there were significantly fewer ED visits, admissions that were unplanned (“urgent/emergent”), and fewer total patient days of hospitalization. Despite this, the total cost per member per month was higher in the LINCC group and the difference approached statistical significance (*p* = 0.06).

In terms of care at the end of life, almost all measures reflect a lower aggressiveness of care for the LINCC group. Use of IV chemotherapy in the last 2 weeks prior to death was so low as to be clinically insignificant for both groups. The differences in all other metrics except total cost in the last 30 days were statistically significant.

The use of hospice among patients who died was examined in two ways (Table 3): comparing patients in the control group with those who received LINCC services and comparing those who received 0 or 1 palliative care visit with those who received more than 1 visit. While the length of stay in hospice was not different, a significantly greater proportion of each group (LINCC: 36.6% vs control: 25.4%) were enrolled in hospice prior to death. However, the receipt of formal palliative consultation visits was not associated with either referral to hospice or length of stay in hospice.

**Table 3:** Hospice use among patients who died^a^

**Variable**	**Control (*n* = 390)**	**LINCC (*n* = 688)**	***p*-value**
Percent of decedents admitted to hospice	25.4%	36.6%	< .001
Hospice average length of stay	(*n* = 94) 39.6 days	(*n* = 221) 36.4 days	.65
**Variable**	**0–1 PC visits**	** > 1 PC visit**	***p*-value**
Hospice average length of stay (SD)	(*n* = 142) 35.6 (52.0) days	(*n* = 117) 34.9 (48.7) days	.92
Hospice average length of stay (with covariates)	(*n* = 122) 37.5 days	(*n* = 99) 34.5 days	.63

^a^Cost outliers not removed

Finally, we examined quality of life measures over time (T1 = enrollment, T2 = 3 months; T3 = 6 months) using total scores from the FACT-G. Because the control group had no data measuring quality of life, we compared LINCC patients with an historical group from an earlier study of advanced cancer patients. That study enrolled patients with newly diagnosed advanced gastrointestinal, lung, and gynecologic cancers and provided supportive services from a team of advanced practice nurses (APNs), social workers, and a spiritual care counselor; although the APNs focused on symptom management, palliative care referrals were not provided.

**Fig. 1:**
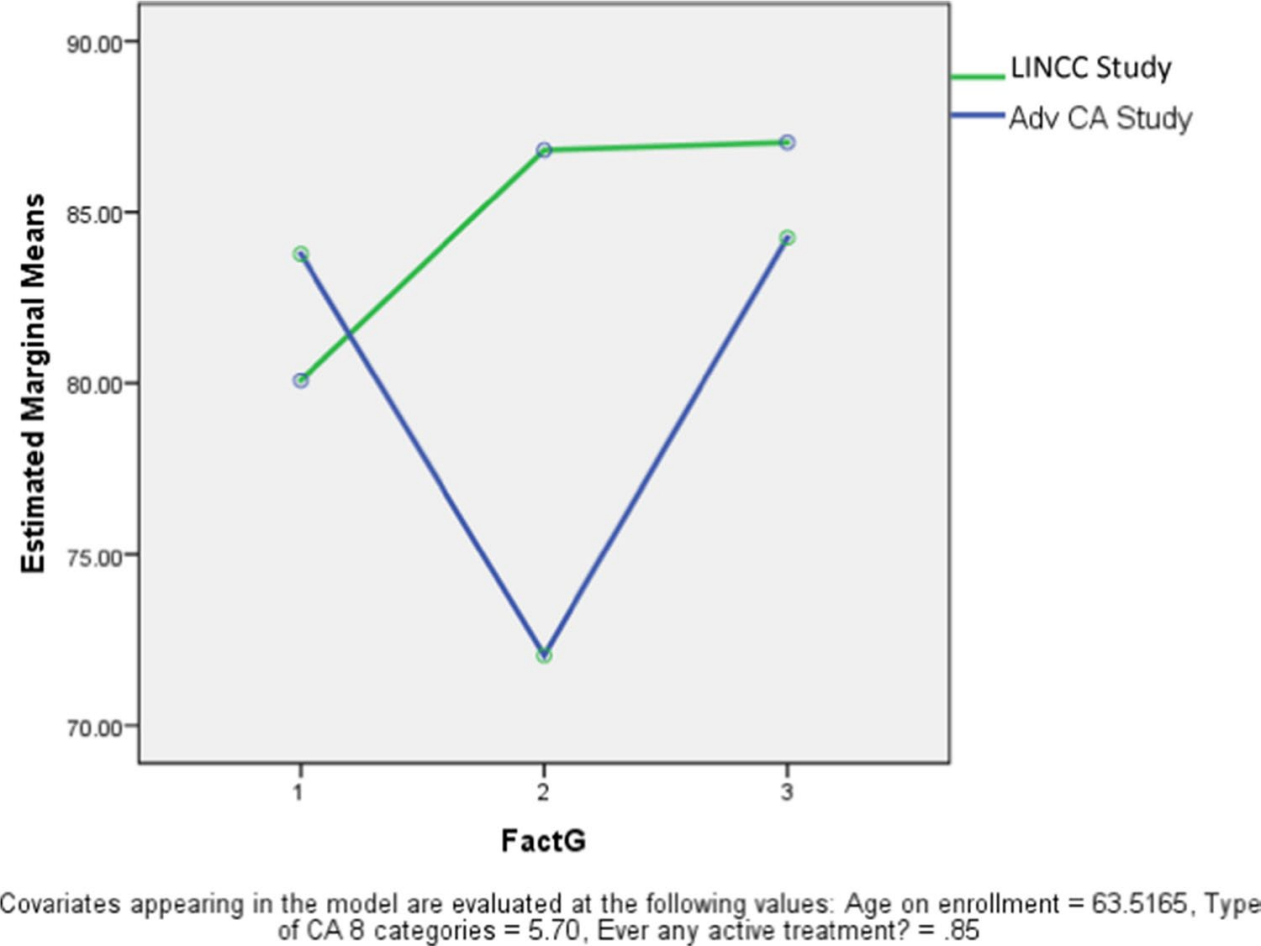
Comparison of FACT-G scores on enrollment among patients from the Advanced Cancer Study (*n* = 216) and newly diagnosed patients from LINCC (*n* = 26)

In order to control for variations associated with time since diagnosis and type of cancer, we included only LINCC patients with GI, lung, and gynecologic cancers who had been diagnosed within the previous 6 months. While this reduced the sample size for LINCC to only 26, there was a significant difference in the pattern of change over time (see Fig. 1). While the LINCC patients had a slightly lower total FACT-G score at enrollment, they had a marked improvement by time 2, which was significantly different than the comparison study group and this difference was maintained at time 3.

## Discussion

Palliative care services have become increasingly available over the past two decades. Recent reports suggest that half of all hospitals with > 50 beds, and as much as 90% of hospitals with > 300 beds, now have some type of palliative care program [18, 19]. As programs have grown, so too has the evidence base of the improvements in patient outcomes associated with palliative care. However, most reports document outcomes either within very specific, disease-oriented categories (e.g., stroke, heart failure, and lung cancer) or have been limited in the type of outcomes measured [20–22]. This study is one of the first to report on an effort to fully integrate care coordination and palliative care in standard cancer care for patients with any type of advanced stage disease, reporting on cost outcomes, quality of life, and quality of end-of-life care metrics.

One of the challenges in evaluating palliative care programs is the heterogeneity of the programs. Some programs are limited to one provider who is available to consult when requested, while others, with the benefit of an interdisciplinary team, can provide spiritual support, social services, and other supportive interventions such as music or art therapy. Some care only for inpatients, while others also follow patients in the outpatient setting. Despite these differences, the importance of demonstrating financial sustainability with favorable patient-centered care and outcomes is common to all.

The finding that total costs were not reduced in the LINCC group, despite savings in end-of-life resource use and reduced urgent admissions and inpatient days, is puzzling. We examined categories of charges such as inpatient services and pharmacy, both as statistical covariates and dependent variables, and though we noted some differences, we cannot fully explain the larger total costs in patients cared for by the LINCC team. Here, the simplest explanation may be the most important. By increasingly accepting the sickest patients into the program (refer to HCC score in Table 1), we may have introduced a selection bias which resulted in under-representation of high-cost therapies and other services in our control population. Still, the results for healthcare costs at end-of-life are promising and warrant further study (Table 3).

Our program was a pragmatic demonstration of a care coordination program that included, as formal elements, systematic and routine symptom assessment with early referral to palliative care, integrated within a large academic practice across a broad range of specific cancer types and at several sites of service. As such, it lacked the rigid controls of clinical trials but allowed us the flexibility to modify the operation of the program as we evaluated barriers and facilitators. For example, the nurse care coordinators were able to interact with the primary teams in varying ways, accommodating preferences of each team. While we were constrained in full implementation of the program at some times over the 3 years because of staffing shortages, this matched real-world conditions likely to occur in the context of non-study operations.

Demonstration projects, much like true pragmatic trials, lack explanatory power but can provide valuable insights contributing to generalizability [23]. Observations from both routine data, such as patient-reported assessments, and anecdotal feedback from program staff and providers can feed data-driven decisions. This can position organizations for success under value-based reimbursement if the organization has learning systems in place to understand the information and subsequently mobilize the organization through quality and process improvement initiatives. There were several important lessons learned from our project.

First, from a cost perspective, our results do not immediately support adopting a similar program that targets all advanced cancer patients regardless of prognosis. Still, given the encouraging results of improved quality at end of life among patients receiving LINCC services, focusing such a program on patients with limited life expectancy appears to be a more sustainable design. This does not negate the recommendation for earlier incorporation of palliative care in cancer but does suggest that focusing on patients who are most likely to be facing end-of-life decisions in the next 6–12 months may be more economically sustainable. The question of dosing, how early such a program may need to intervene with terminal patients to achieve favorable results on cost and patient quality of life, remains unanswered.

Second, while we do not have data supporting this, it was clear from the outset of the project that fully integrating the care coordinators within the primary oncology team had multiple advantages. This approach required a significant investment of staff time, but being part of routine reviews of clinic schedules and team discussions of patient treatment plans enabled the care coordinators to build credibility with clinicians, facilitate patient referrals, incorporate palliative care recommendations, and prioritize patient needs through better communication. There were numerous occasions in which the care coordinator was able to intervene early in a patient problem, preventing avoidable ER visits or improving symptom management.

Third, the flexibility of the program allowed for an added focus on improving the infrastructure. It became apparent as the program went on that the documentation, and thus communication, of goals of care discussions was inconsistent and quite variable. Under the auspices of the program, a new form for the electronic medical record was designed and implemented. Though not part of the original features of the demonstration project, the addition of this form to routine care was a significant improvement in the timing and adequacy of communication around the sensitive topic of end-of-life decisions and plans.

Finally, implementing a system-wide program such as LINCC requires deliberative alignment with existing system strategic planning, leadership support and investment of resources, and a commitment to system learning. The positive results in terms of overall quality of life, reduced cost at end of life, and improved quality of care at end of life support the adoption of similar programs, with a more focused effort to target patients with a defined poor prognosis.

## Data Availability

N/A

## Code availability

N/A
